# Enzyme self-label-bound ATTO700 in single-molecule and super-resolution microscopy†

**DOI:** 10.1039/d2cc04823j

**Published:** 2022-11-21

**Authors:** Michael Trumpp, Anna Oliveras, Hannes Gonschior, Julia Ast, David J. Hodson, Petra Knaus, Martin Lehmann, Melissa Birol, Johannes Broichhagen

**Affiliations:** Leibniz-Forschungsinstitut für Molekulare Pharmakologie (FMP) Robert-Rössle-Str. 10 13125 Berlin Germany broichhagen@fmp-berlin.de; Freie Universität Berlin, Institute of Chemistry and Biochemistry – Biochemistry Thielallee 63 14195 Berlin Germany; Berlin Institute of Medical Systems Biology (BIMSB), Max Delbrück Center for Molecular Medicine Hannoversche Str. 28 10115 Berlin Germany Melissa.Birol@mdc-berlin.de; Institute of Metabolism and Systems Research (IMSR), and Centre of Membrane Proteins and Receptors (COMPARE), University of Birmingham Birmingham UK; Oxford Centre for Diabetes, Endocrinology and Metabolism (OCDEM), NIHR Oxford Biomedical Research Centre, Churchill Hospital, Radcliffe Department of Medicine, University of Oxford Oxford OX3 7LE UK

## Abstract

Herein, we evaluate near-infrared ATTO700 as an acceptor in SNAP- and Halo-tag protein labelling for Förster Resonance Energy Transfer (FRET) by ensemble and single molecule measurements. Microscopy of cell surface proteins in live cells is perfomed including super-resolution stimulated emission by depletion (STED) nanoscopy.

## Abstract 2

Near-infrared ATTO700 is evaluated as an acceptor in SNAP- and Halo-tag protein labelling for ensemble and single molecule Förster Resonance Energy Transfer (FRET) and in stimulated emission by depletion (STED) super resolution microscopy.

## Graphical abstract

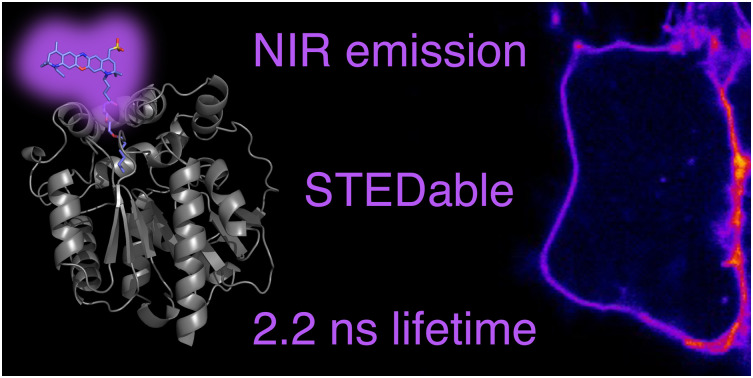

In recent years, the chemical synthesis and photophysical testing of new fluorescent dyes has experienced a renaissance, aiming for enhanced brightness and prolonged photostability.^1^ As a consequence, less attention has been paid to “older” fluorophores, which remain under-evaluated for their performance in advanced imaging. We therefore revisited ATTO700, which displays desirable properties including high extinction coefficient (*ε* = 120 000 M^−1^ cm^−1^) and near-infrared (NIR) absorption and emission (*λ*_Exc/Em_ = 700/716 nm) for less photodamage and better tissue penetration. Yet other parameters can be considered moderate, such as quantum yield (*Φ* = 25%) and lifetime (*τ* = 1.6 ns), according to the manufacturer. While ATTO700 has been used successfully for single-molecule imaging,^2^ blinking properties^3^ and stochastic optical reconstruction microscopy (STORM),^4^ our aim was to evaluate its performance for other imaging techniques, in particular those where NIR dyes are scarce. We hypothesized that ATTO700 may perform better when bound to a protein surface in comparison to its freely-diffusing congeners, since most dyes are environmentally sensitive. Indeed, in 2010, a study from Stöhr *et al.* observed that ATTO700, when fused to a *O*^6^-benzylguanine (BG) substrate for the self-labelling SNAP26b-tag, showed a turn-on of ∼30-fold upon reaction.^5^ This was attributed to the guanine group, which quenches the fluorophore by a photoinduced electron transfer (PeT) mechanism and is lost upon covalent linkage with SNAP. As a start, we aimed to reproduce these results, and indeed found a turn-on of 2.9-fold, 5.9-fold and 8.3-fold in absorbance, quantum yield and fluorescent emission, respectively, when BG-ATTO700 reacted with a recombinantly expressed SNAP-Halo construct (Fig. 1A, *Φ*_BG-ATTO700_ = 3.5 ± 0.4%; *Φ*_SNAP:ATTO700-Halo_ = 20.8 ± 0.0%, see ESI†). All labelled constructs were validated using mass spectrometry on the full-length protein (see ESI†). Since the PeT mechanism cannot occur before the Halo-tag reaction, due to the leaving group being a simple chloride anion, we synthesized its substrate chloroalkane (CA-)ATTO700, before repeating experiments. As expected, no strong unquenching was observed, although we did notice enhanced absorbance, quantum yield and fluorescent emission by 1.7-fold, 1.1-fold and 1.6-fold, respectively, when using the SNAP-Halo construct (Fig. 1B, *Φ*_CA-ATTO700_ = 21.9 ± 0.5%; *Φ*_SNAP-Halo:ATTO700_ = 24.7 ± 0.2%), which we attribute to dye-protein surface interactions. Comparing the raw emission and quantum yield, we observed slightly higher values when ATTO700 was bound to Halo compared to SNAP. Encouraged by these results, we first set out to test the SNAP-Halo construct in Förster Resonance Energy Transfer (FRET) experiments, a phenomenon widely used in the communities to measure distances and molecular motions of labelled proteins. Choosing Halo:ATTO700 as the acceptor molecule, we wanted to compare a small set of far-red dyes to serve as FRET donors. Accordingly, the SNAP-Halo was labelled with CA-ATTO700 and either BG-Sulfo646,^6^ BG-SulfoCy5,^7^ BG-JF_646_^8^ or BG-SiR-d12^9^ (Fig. 1C, see ESI†). Using this approach, we observed different efficiencies of energy transfer, described by ratios between 1.2–2.1 (calculated as FRET/donor = *λ*_710–730 nm_/*λ*_660–680 nm_), with JF_646_ and SiR-d12 serving as the best FRET donors for ATTO700. To visualize the SNAP-Halo construct, we used ColabFold^10^ on the protein sequence and modelled SiR-d12 and ATTO700 using PyMOL (Fig. 2A). Without further energy optimization we measured a distance *d* ∼4 nm between the fluorophores. Next, using fluorescence lifetime spectroscopy, we were able to quantitatively report on the change in the fluorescence lifetime decay of donor only (*τ*_D_) and donor–acceptor samples (*τ*_DA_). Here, time-correlated single-photon counting was performed using pulsed excitation to reconstruct *τ*, and decays were fitted to an exponential function to model life-time. We first aimed to compare *τ*_BG-ATTO700_ to *τ*_SNAP:ATTO700-Halo_, however, the dim nature of BG-ATTO700 prevented meaningful fitting, thus we only report on *τ*_SNAP:ATTO700-Halo_ = 2.2 ns (Fig. 2B). This is in agreement with lifetimes acquired on the ensemble level (*τ*_SNAP:ATTO700_ = 2.2 ns).^5^ However, it was possible to acquire and compare lifetimes for *τ*_CA-ATTO700_ and *τ*_SNAP-Halo:ATTO700_ (Fig. 2B). With a reported lifetime *τ*_ATTO700_ = 1.6 ns, we found *τ*_CA-ATTO700_ = 1.9 ns, and decay time was also increased when bound to Halo (*τ*_SNAP-Halo:ATTO700_ = 2.2 ns). This increase of lifetime by ∼20% makes ATTO700 an interesting alternative to fluorescent proteins in the NIR regime, with STED near-infrared fluorescent protein (SNIFP) serving as an example (*τ* = 0.63 ns).^11^ After assessing acceptor performance, we next correlated the constructs using ensemble FRET measurements, and also observed reduced *τ*_D_ for various donors in the SNAP-Halo construct when CA-ATTO700 acts as acceptor (*cf.*Fig. 1). In these experiments, the donor probes Sulfo646, JF_646_, and SiR-d12 were excited and changes in *τ* in the absence and presence of Halo:ATTO700 as an acceptor are reported in Fig. 2C. As expected for all pairs tested, a reduced *τ* is observed. We calculated FRET efficiencies (*E*_FRET_) for the pairs, with SiR-d12 showing the highest *E*_FRET_ = 28%. This leads to a dye-dye distance of 8.8 nm (assuming *κ*^2^ = 2/3), with a calculated Förster radius *R*_*0*_ = 7.3 nm. This is in stark contrast to the static and solvent-free protein model (Fig. 2A) and shows that *in silico* predicted structures do not necessarily reflect dynamic behavior of proteins that contain flexible amino acid linkers on the single molecule level. In this case, the predicted folding led to SNAP–Halo surface interactions, which may be absent in solution. Nonetheless, the results are still in agreement with ensemble FRET measurements where SiR-d12 displayed the largest ratio alongside JF_646_. Furthermore, we find that SulfoCy5 as a donor did not show satisfying *τ* both in the absence and presence of Halo:ATTO700, however, this observation shows crosstalk-free donor detection. It has previously been reported that SulfoCy5 has lifetimes between 1.0 to 2.3 ns depending on its environment,^12^ and therefore makes it less suitable for our single molecule experiments. To conclude, rhodamine structures should be regarded as scaffolds of choice when FRET is performed with ATTO700 as an acceptor.

**Fig. 1:**
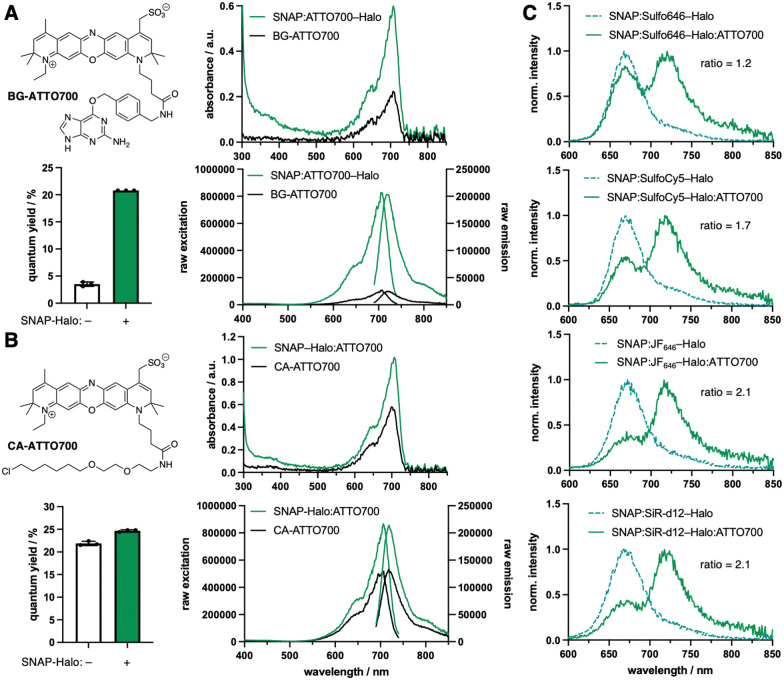
ATTO700 in and SNAP- and Halo-tag protein labelling. (A) Structure of BG-ATTO700 and its properties when bound to a SNAP-Halo-tag construct including changes in absorbance, quantum yield and fluorescence excitation/emission. (B) As for A but with CA-ATTO700. (C) FRET measurements on a SNAP-Halo-tag construct with ATTO700 serving as the acceptor, while the donor was either Sulfo646, SulfoCy5, JF_646_ or SiR-d12, with ratio changes from top to bottom.

**Fig. 2:**
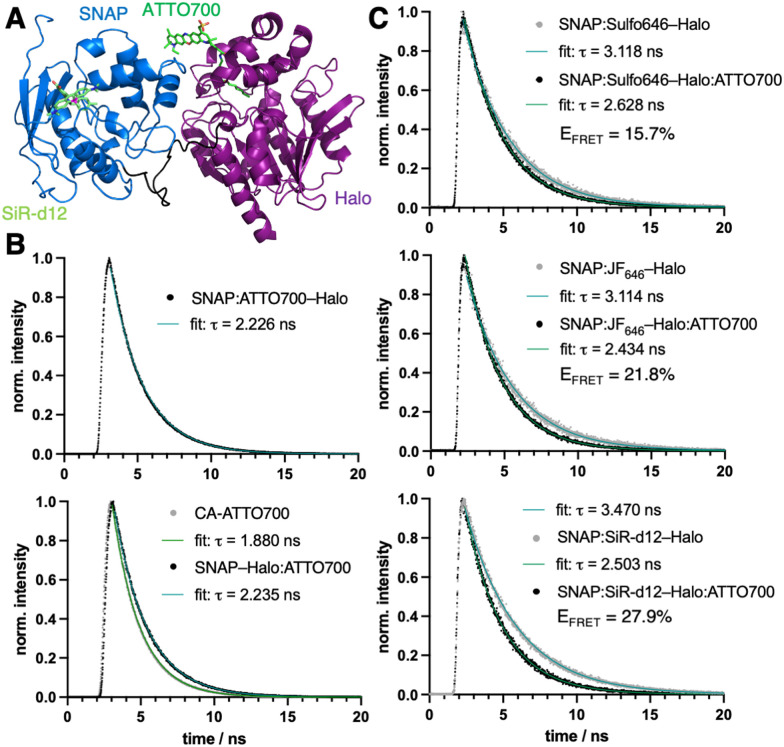
Single-molecule characterization with ATTO700. (A) Structural model of SNAP:SiR-d12–Halo:ATTO700. (B) Lifetime measurements of acceptor only bound to SNAP (top) and in solution and Halo-bound (bottom). (C) Fluorescence lifetime spectroscopy of donor only (from top to bottom: Sulfo646, JF_646_ and SiR-d12) and with ATTO700 as an acceptor allows accurate calculation of FRET efficiencies (*E*_FRET_).

We next explored the utility of ATTO700 for live-cell imaging. First, using the WST-1 assay we determined that, at the concentrations used, BG-ATTO700 and CA-ATTO700 do not affect HEK293 cell viability (Fig. S1, ESI†). Next, to check that ATTO700 is cell-impermeable, we transfected HEK293 cells with a construct^6^ (Fig. 3A) that places the SNAP-tag on the extracellular side of the plasmalemma due to an added transmembrane (TM) domain, while the Halo-tag resides in the intracellular space (SNAP_f_-TM-Halo). SNAP_f_-TM-Halo labelling resulted in only weak signals using either BG-ATTO700 or CA-ATTO700, which was unexpected for the extracellular SNAP_f_-tag (since *in vitro* labelling was successful, *vide supra*), but anticipated for the intracellular Halo-tag. Confirming expression, we labelled the construct with a second color, *i.e.* either permeable CA-JF_549_ or impermeable BG-Sulfo549 (Fig. S2A, ESI†), and confirmed sufficiently high expression levels. We then turned to the Halo-TM-SNAP_f_ construct that presents the labelling tags the other way around across the plasma membrane. As expected, we did not observe any intracellular SNAP_f_-tag labelling, but clear and strong Halo-tag surface staining (Fig. 3B) with the respective ATTO700s. Again, expression levels were controlled with impermeable CA-Sulfo549 or permeable BG-JF_549_ (Fig. S2B, ESI†).

**Fig. 3:**
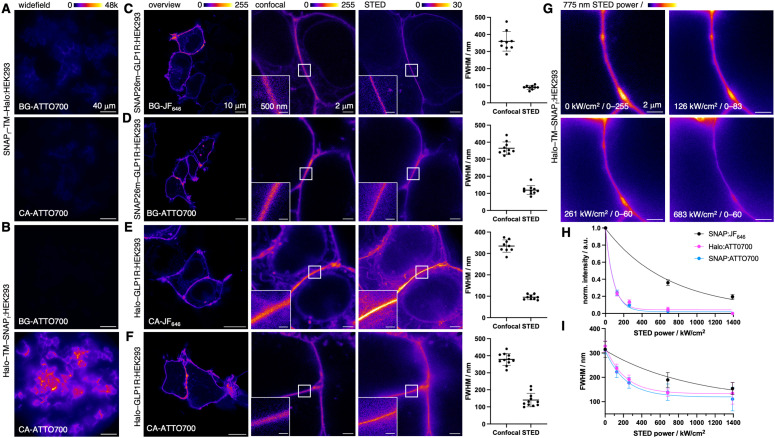
Live cell microscopy using ATTO700. (A) Widefield imaging of live SNAPf-TM-Halo:HEK293 cells incubated with either BG-ATTO700 (top) or CA-ATTO700 (bottom). (B) Widefield imaging of live Halo-TM-SNAPf:HEK293 cells incubated with either BG-ATTO700 (top) or CA-ATTO700 (bottom). (C) Imaging of live SNAP26m-GLP1R:HEK293 cells labelled with BG-JF_646_ showcasing from left to right an overview, a zoomed-in image in confocal and STED mode, with full width at half-maximum calculations of membrane contacts. (D) As for C but labelled with BG-ATTO700. (E) Imaging of live Halo-GLP1R:HEK293 cells labelled with CA-JF_646_. (F) As for E but labelled with CA-ATTO700. (G) Representative images of membrane contact sites of transfected Halo-TM-SNAP_f_ cells labelled with CA-ATTO700 under different STED depletion intensities. (H) Saturation intensity *I*_sat_ of SNAP and Halo-bound ATTO700 including SNAP-bound JF_646_. (I) As in H but for depletion efficiency Eff_Depl_. *I*_sat_ and Eff_Depl_*vs.* 775 nm depletion power was measured from three independent membrane contact regions with constant excitation but varying 775 nm depletion power.

We next turned to the glucagon-like peptide-1 receptor (GLP1R), a class B G protein-coupled receptor (GPCR), involved in glucose-dependent insulin secretion. While GLP1R is well-characterized, there is still interest in its localization and trafficking, which might explain differences in responses to the various therapeutic ligands.^13,14^ Cell impermeable NIR dyes that preferentially label surface GLP1R are warranted for its investigation and biology applications. We therefore tested ATTO700 for its ability to label SNAP and Halo-tagged GLP1R. To this end, HEK293 cells were transfected with SNAP26m-GLP1R before labelling with BG-JF_646_ (Fig. 3C) or BG-ATTO700 (Fig. 3D). Employing confocal microscopy, we obtained clear images for cell surface labelling, and unlike the SNAP_f_-TM-Halo construct, SNAP26m-GLP1R was successfully ATTO700-labelled (*cf.*Fig. 3A *vs*. D).

We hypothesized that this puzzling behavior might arise from either: (1) proximity of the tags to the cell surface causing steric hindrance or repulsion from the negatively charged surface, or (2) the slightly different amino acid sequence of the tag. To test these hypotheses, we cloned a SNAP26m-TM-Halo construct (see ESI†) and reperformed the labelling experiment (Fig S2A, ESI†). Presence of only weak signals would demonstrate membrane proximity effects, while comparable labelling to SNAP26m-GLP1R would account for tag bias. Indeed, we detected similar signal intensities when comparing SNAP_f_-TM-Halo and SNAP26m-TM-Halo, supporting the role for steric hindrance and/or repulsion in signal loss. This observation is further supported by the distance of SNAP26m *N*-terminally fused to GLP1R, since the ectodomain of the GPCR separates the tag from the surface. Why is this not the case for Halo-TM-SNAP? Consulting crystal structures and exit channels of SNAP and Halo, the *C*-terminus is considerably closer to the cell membrane for SNAP (Fig. S3, ESI†). This requires the dye to get closer to the cellular surface to react covalently. Ultimately, this finding demonstrates the need to carefully validate different systems.

We next turned to STED super-resolution imaging to probe if ATTO700 is amenable to the high laser powers used to circumvent Abbe's Law, since a de-excitation beam at *λ* = 775 nm is used. In both cases, the diffraction limit was broken, and line scans with a full width at half maximum (FWHM) were obtained at ∼100 nm for membrane contact sites (Fig. 3D), with comparable performance to JF_646_ (Fig. 3C). Similar results were obtained when HEK293 cells were transfected with Halo-GLP1R followed by labelling with CA-JF_646_ (Fig. 3E) and CA-ATTO700 (Fig. 3F). While both dyes were STEDable, we noticed more pronounced intracellular labelling using JF_646_, while ATTO700 was restricted to the surface (*cf.*Fig. 3E and F), in our case a desirable trait for examination of surface GPCRs. We aimed to push the boundaries of STED imaging, by looking at SNAP- and Halo-tagged Tubb5^9^ and claudin10a,^15^ where structures have constant diameters of 25 and 10 nm, respectively (Fig. S4, ESI†). Although signals were detected in post-fix labelled SNAP-Tubb5, Halo-Tubb5 and SNAP-claudin10a expressing COS7 cells, these were not amenable to STED nanoscopy due to their low signal intensities. Thus, while interrogation of intracellular targets remains limited with ATTO700, they could in the future be addressed if the phenoxazine-linked sulfonate is masked or erased to allow cell permeability (depending on the BG substrate).^16^ Nevertheless, these experiments demonstrate the applicability of ATTO700 to super-resolution imaging on the cell surface, with the dye comparing favorably to NIR fluorescent STEDable proteins such as SNIFP^11^ or iRFP680.^17^ It should be noted that super-resolution images of ATTO700 were acquired with 10–15% 775 nm STED power (*cf.* 40–60% for JF_646_) for efficient depletion. Therefore, ATTO700 is prone for less photobleaching as de-excitation powers usually outweigh excitation intensities by orders of magnitudes. To further characterize this, we recorded images with different depletion powers (Fig. 3G) and plotted against fluorescence intensity and FWHM to find the depletion efficiency (Eff_Depl_ (Halo:ATTO700) = 145.3 kW cm^−2^; Eff_Depl_ (SNAP:ATTO700) = 151.3 kW cm^−2^) (Fig. 3H) and saturation intensity (*I*_sat_ (Halo:ATTO700) = 57.5 kW cm^−2^; *I*_sat_ (SNAP:ATTO700) = 61.0 kW cm^−2^) (Fig. 3I), respectively. All values compare favorably to SBG-JF_646_ surface labelled SNAP-tags (Eff_Depl_ = 653.8 kW cm^−2^; *I*_sat_ = 479.9 kW cm^−2^) (Fig. 3H, I and see also Fig. S5, ESI†), demonstrating the advantage of lower light intensities for deeper tissue imaging with less phototoxicity.

In summary, we have revisited the NIR dye ATTO700, finding that its photophysical performance is drastically enhanced when bound to SNAP- and Halo-tags, and demonstrate its applicability in single-molecule and super-resolution microscopy. Since fluorophores with spectral characteristics beyond 700 nm are scarce when compared to their green, red and far-red stablemates, we anticipate that ATTO700 will find more applications in molecular and cellular studies for surface exposed SNAP- and Halo-tagged proteins.

M. T. and J. B. performed chemical synthesis and characterization, protein labelling and ensemble measurements. A. O., M. B. and J. B. recorded single molecule spectroscopy. H. G. and M. L. performed STED nanoscopy. J. A., D. J. H. and P. K. provided reagents, protocols and resources. M. B. and J. B. conceived and supervised the study and wrote the manuscript with input from all authors. We thank Kilian Roßmann, Pascal Poc, Ramona Birke, Rozemarijn van der Veen for assistance, Kristin Kemnitz-Hassanin and Christian P. R. Hackenberger (all FMP) and Jana Roßius (BIMSB-MDC) for support.

P. K. was supported by DFG (SFB1444). This work was supported by the Sonnenfeld Foundation with a stipend to H. G. and a grant from the DFG to M. L. (GRK2318/TJ-Train A4). D. J. H. was supported by MRC (MR/N00275X/1 and MR/S025618/1) Project and Diabetes UK (17/0005681) Project Grants, as well as an ERC Frontier Research Guarantee Grant (EP/X026833/1). This project has received funding from the European Research Council (ERC) under the European Union's Horizon 2020 research and innovation programme (Starting Grant 715884 to D. J. H.) and under the European Union's Horizon Europe Framework Programme (deuterON, Grant agreement no. 101042046 to J. B.). The research was funded by the National Institute for Health Research (NIHR) Oxford Biomedical Research Centre (BRC).

## Conflicts of interest

There are no conflicts to declare.

## Supplementary Material

CC-058-D2CC04823J-s001
