# Supplementary material for: Enzyme self-label-bound ATTO700 in single-molecule and super-resolution microscopy
Source: Chem Commun (Camb). 2022 Nov 21;58(99):13724–7. doi: 10.1039/d2cc04823j (PMC9745879; doi:10.1039/d2cc04823j)
Supplement: CC-058-D2CC04823J-s001 [file CC-058-D2CC04823J-s001.pdf]

Electronic Supplementary Material (ESI) for Chemical Communications.
This journal is © The Royal Society of Chemistry 2022

Supplemental Information for:

Enzyme self-label-bound ATTO700 in single molecule and super-resolution microscopy

Michael Trumpp[1,2], Anna Oliveras[3], Hannes Gonschior[1], Julia Ast[4], David J. Hodson[4,5], Petra Knaus[2], Martin Lehmann[1], Melissa Birol[3,*] and Johannes Broichhagen[1,*]

1. Leibniz-Forschungsinstitut für Molekulare Pharmakologie (FMP), Robert-Rössle-Str. 10, 13125 Berlin, Germany.
2. Freie Universität Berlin, Institute of Chemistry and Biochemistry - Biochemistry, Thielallee 63, 14195 Berlin, Germany
3. Berlin Institute of Medical Systems Biology, Max Delbrück Center for Molecular Medicine, Hannoversche Str. 28, 10115 Berlin, Germany.
4. Institute of Metabolism and Systems Research (IMSR), and Centre of Membrane Proteins and Receptors (COMPARE), University of Birmingham, Birmingham, UK.
5. Oxford Centre for Diabetes, Endocrinology and Metabolism (OCDEM), NIHR Oxford Biomedical Research Centre, Churchill Hospital, Radcliffe Department of Medicine, University of Oxford, Oxford, OX3 7LE, UK.

To whom correspondence should be addressed:
Melissa.birol@mdc-berlin.de and broichhagen@fmp-berlin.de

Table of Contents

1. General 3

2. Synthesis 5

2.1. (11-(4-((4-(((2-Amino-9H-purin-6-yl)oxy)methyl)benzyl)amino)-4-oxobutyl)-1-ethyl-2,2,4,10,10-pentamethyl-10,11-dihydro-2H-dipyrido[3,2-b:2',3'-i]phenoxazin-1-ium-8-yl)methanesulfonate (BG-ATTO700) 5

2.2. (11-(4-((2-(2-((6-Chlorohexyl)oxy)ethoxy)ethyl)amino)-4-oxobutyl)-1-ethyl-2,2,4,10,10-pentamethyl-10,11-dihydro-2H-dipyrido[3,2-b:2',3'-i]phenoxazin-1-ium-8-yl)methanesulfonate (CA-ATTO700) 6

3. NMR spectra 7

3.1. (11-(4-((4-(((2-Amino-9H-purin-6-yl)oxy)methyl)benzyl)amino)-4-oxobutyl)-1-ethyl-2,2,4,10,10-pentamethyl-10,11-dihydro-2H-dipyrido[3,2-b:2',3'-i]phenoxazin-1-ium-8-yl)methanesulfonate (BG-ATTO700) 7

3.2. (11-(4-((2-(2-((6-Chlorohexyl)oxy)ethoxy)ethyl)amino)-4-oxobutyl)-1-ethyl-2,2,4,10,10-pentamethyl-10,11-dihydro-2H-dipyrido[3,2-b:2',3'-i]phenoxazin-1-ium-8-yl)methanesulfonate (CA-ATTO700) 8

4. Protein labelling 9

4.1. SNAP–Halo construct[2] 9

4.2. SNAP–Halo expression and purification 9

4.3. Protein labelling 9

4.3.1. For turn-on measurements 9

4.3.2. For FRET measurements 10

4.4. SNAP–Halo mass spectrometry[3] 10

5. SNAP26m-TM-Halo Cloning 11

5.1. Construct 11

5.2. Primer 11

5.3. Protocol 11

6. Cell culture 12

6.1. Culture protocols 12

6.2. WST-1 viability assay 12

7. Microscopy 13

7.1. Widefield, confocal and STED 13

7.2. Single molecule imaging, fluorescence life-time 13

8. Supplementary Figures 14

9. Supplementary Schemes 19

10. References 20

1. General

All chemical reagents and anhydrous solvents for synthesis were purchased from commercial suppliers (Sigma-Aldrich, Fluka, Acros, Fluorochem, TCI) and were used without further purification if not stated otherwise. Commercial coumarin 461 and methylene blue were HPLC purified before concentration assessment and measuring photophysical properties to ensure similar purity and composition as their synthesizes, deuterated counterparts. BG-TMR and BG-SiR were described before.[1]

NMR spectra were recorded in deuterated solvents on a Bruker AV-III spectrometers using either a cryogenically cooled 5 mm TCI-triple resonance probe equipped with one-axis self-shielded gradients or room-temperature 5 mm broadband probe and calibrated to residual solvent peaks (^{1}H/^{13}C in ppm): MeOD-d_4 (3.30). Multiplicities are abbreviated as follows: s = singlet, d = doublet, t = triplet, q = quartet, p = pentet, h = heptet, br = broad, m = multiplet. Coupling constants *J* are reported in Hz. Spectra are reported based on appearance, not on theoretical multiplicities derived from structural information.

UPLC-UV/Vis for purity assessment was performed on an Agilent 1260 Infinity II LC System equipped with Agilent SB-C18 column (1.8 µm, 2.1 × 50 mm). Buffer A: 0.1% FA in H_2O Buffer B: 0.1% FA acetonitrile. The typical gradient was from 10% B for 0.5 min → gradient to 95% B over 5 min → 95% B for 0.5 min → gradient to 99% B over 1 min with 0.8 mL/min flow. Retention times (t_R) are given in minutes (min). Chromatograms were imported into Graphpad Prism8 and purity was determined by calculating AUC ratios.

Preparative or semi-preparative HPLC was performed on an Agilent 1260 Infinity II LC System equipped with columns as followed: preparative column –Reprospher 100 C18 columns (10 µm: 50 x 30 mm at 20 mL/min flow rate; semi-preparative column – 5 µm: 250 x 10 mm at 4 mL/min flow rate. Eluents A (0.1% TFA in H_2O) and B (0.1% TFA in MeCN) were applied as a linear gradient. Peak detection was performed at maximal absorbance wavelength.

High resolution mass spectrometry was performed on an Agilent Technologies 6230 series accurate mass TOF LC-MS linked to an Agilent Technologies 1290 Infinity Series machine with a Thermo Accucore™ RP-MS column, 2.6 µm pore size, 30 × 2.1 mm, and a 3 min gradient from 5 to 99% aqueous MeCN with 0.1% TFA and MeCN with 0.1% TFA. flow rate: 0.8 mL/min; UV-detection: 220 nm, 254 nm, 300 nm.

Intact proteins were analyzed using a Waters H-class instrument equipped with a quaternary Solvent manager, a Waters sample manager-FTN, a Waters PDA detector and a Waters column manager with an Acquity UPLC protein BEH C4 column (300 Å, 1.7 µm, 2.1 mm x 50 mm). Proteins were eluted with a flow rate of 0.3 mL/min at a column temperature of 80 °C. The following gradient was used: A: 0.01% FA in H_2O; B: 0.01% FA in MeCN. gradient 5-95% B from 0-6 min. Mass analysis was conducted with a Waters XEVO G2-XS QTof analyzer. Proteins were ionized in positive ion mode applying a cone voltage of 40 kV. Raw data was analyzed with MaxEnt 1. After deconvolution of the crude spectra, no single or non-labelled SNAP-Halo construct was observed, indicating complete reaction.

Chemical structures of used fluorescent donors:

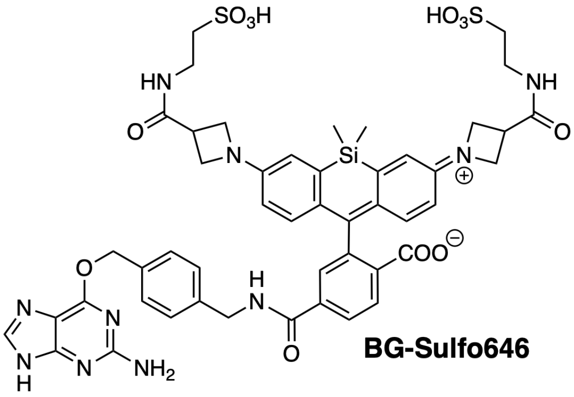

BG-Sulfo646

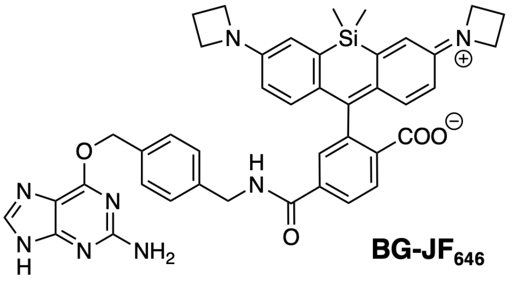

BG-JF$_{646}$

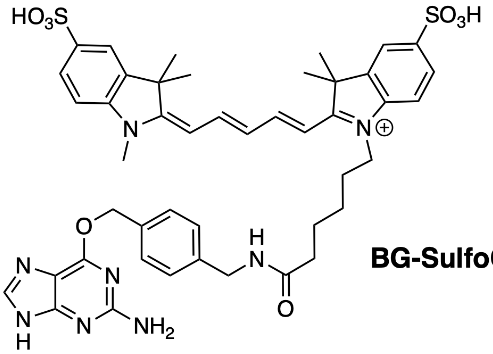

BG-SulfoCy5

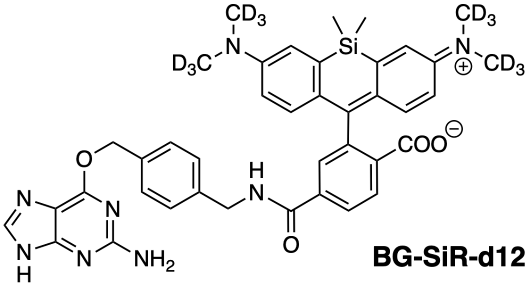

BG-SiR-d12

2. Synthesis

2.1. (11-(4-((4-(((2-Amino-9*H*-purin-6-yl)oxy)methyl)benzyl)amino)-4-oxobutyl)-1-ethyl-2,2,4,10,10-pentamethyl-10,11-dihydro-2*H*-dipyrido[3,2-b:2',3'-i]phenoxazin-1-ium-8-yl)methanesulfonate (BG-ATTO700)

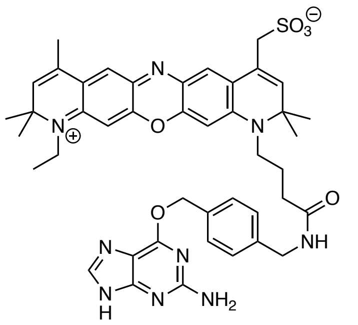

An Eppendorf tube was charged with 500 µg (740 nmol, 1.0 equiv.) ATTO700-COOH (atto-tec, #AD 700-21) dissolved in 200 µL DMF and 6 equiv. DIPEA, before 0.24 mg (0.89 µmol, 1.2 equiv.) of BG-NH_2 and HBTU (0.34 mg, 890 nmol, 1.2 equiv.) were added successively. The reaction mixture was allowed to incubate for 2 h at r.t., before it was quenched with 50 µL HOAc, diluted with a 1:1 mixture of MeCN:H_2O and subjected to RP-HPLC (MeCN:H_2O+0.1% TFA = 10:90 to 90:10 over 45 minutes) to obtain 675 nmol of the desired product after lyophilization in 91% yield.

^{1}H NMR (600 MHz, MeOD-d_4): δ [ppm] = 8.19 (br s, 1H), 7.78 (s, 1H), 7.47 (s, 3H), 7.31 (s, 1H), 6.74 (s, 1H), 6.61 (s, 1H), 5.98 (s, 1H), 5.84 (s, 1H), 5.55 (s, 2H), 4.45 (s, 2H), 4.05 (s, 2H), 3.84 (q, J = 7.0, 2H), 3.52 (m, 2H), 2.41 (t, J = 6.3 Hz, 2H), 2.13 (d, J = 1.1 Hz, 2H), 2.03 (m, 2H), 1.59 (s, 6H), 1.53 (s, 6H), 1.43 (t, J = 7.1 Hz, 3H).

HRMS (ESI): calc. for $C_{43}H_{48}N_9O_6S$ $[M+H]^+$: 818.3443, found: 818.3506.

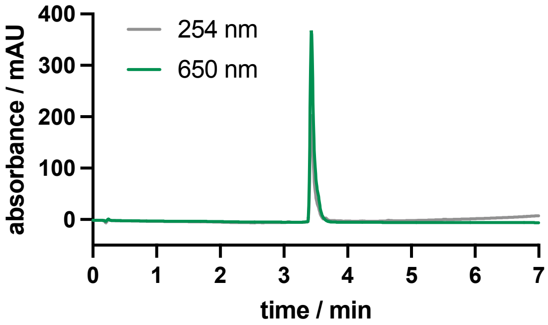

2.2. (11-(4-((2-(2-((6-Chlorohexyl)oxy)ethoxy)ethyl)amino)-4-oxobutyl)-1-ethyl-2,2,4,10,10-pentamethyl-10,11-dihydro-2H-dipyrido[3,2-b:2',3'-i]phenoxazin-1-ium-8-yl)methanesulfonate (CA-ATTO700)

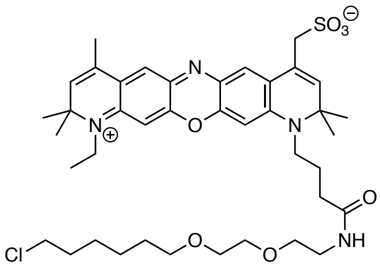

An Eppendorf tube was charged with 0.4 mg (1.24 µmol, 1.7 equiv.) CA-NHBoc and 30 µL neat TFA was added. The reaction mixture was incubated at r.t. for 30 minutes before all volatiles were removed under a gentle stream of nitrogen. The residue was taken up in 200 µL DMF and 6 equiv. DIPEA before 500 µg (740 nmol, 1.0 equiv.) ATTO700-COOH (atto-tec, #AD 700-21) and 340 µg (890 nmol, 1.2 equiv.) HBTU were added successively. The reaction mixture was allowed to incubate for 2 h at r.t., before it was quenched with 50 µL HOAc, diluted with a 1:1 mixture of MeCN:H_2O and subjected to RP-HPLC (MeCN:H_2O+0.1% TFA = 10:90 to 90:10 over 45 minutes) to obtain 675 nmol of the desired product after lyophilization in 91% yield.

^{1}H NMR (600 MHz, MeOD-d_4): δ [ppm] = 7.84 (s, 1H), 7.48 (s, 1H), 7.07 (s, 1H) 6.86 (s, 1H), 6.04 (s, 1H), 5.83 (s, 1H), 4.01 (s, 2H), 3.84 (q, J = 7.0 Hz, 2H), 3.71 (t, J = 8.5 Hz, 2H), 3.63–3.60 (m, 4H), 3.58–3.57 (m, 2H), 3.53 (t, J = 6.7 Hz, 2H), 3.45 (m, 4H), 2.44 (t, J = 6.5 Hz, 2H), 2.12 (d, J = 1.1 Hz, 3H), 2.06 (br s, 2H), 1.75–1.71 (m, 2H), 1.61 (s, 6H), 1.58 (s, 6H), 1.57–1.53 (m, 2H), 1.41 (t, J = 7.1 Hz, 2H), 1.38–1.34 (m, 2H).

HRMS (ESI): calc. for $C_{40}H_{56}ClN_4O_7S$ $[M+H]^+$: 771.3553, found: 771.3563.

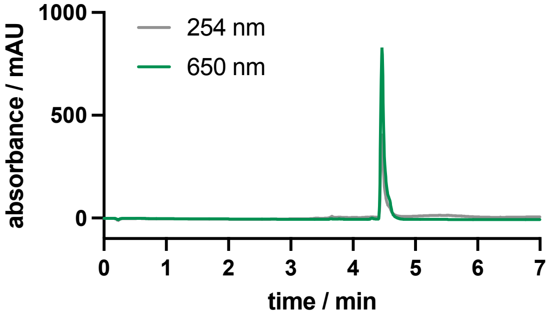

3. NMR spectra

3.1. (11-(4-((4-(((2-Amino-9*H*-purin-6-yl)oxy)methyl)benzyl)amino)-4-oxobutyl)-1-ethyl-2,2,4,10,10-pentamethyl-10,11-dihydro-2*H*-dipyrido[3,2-b:2',3'-i]phenoxazin-1-ium-8-yl)methanesulfonate (BG-ATTO700)

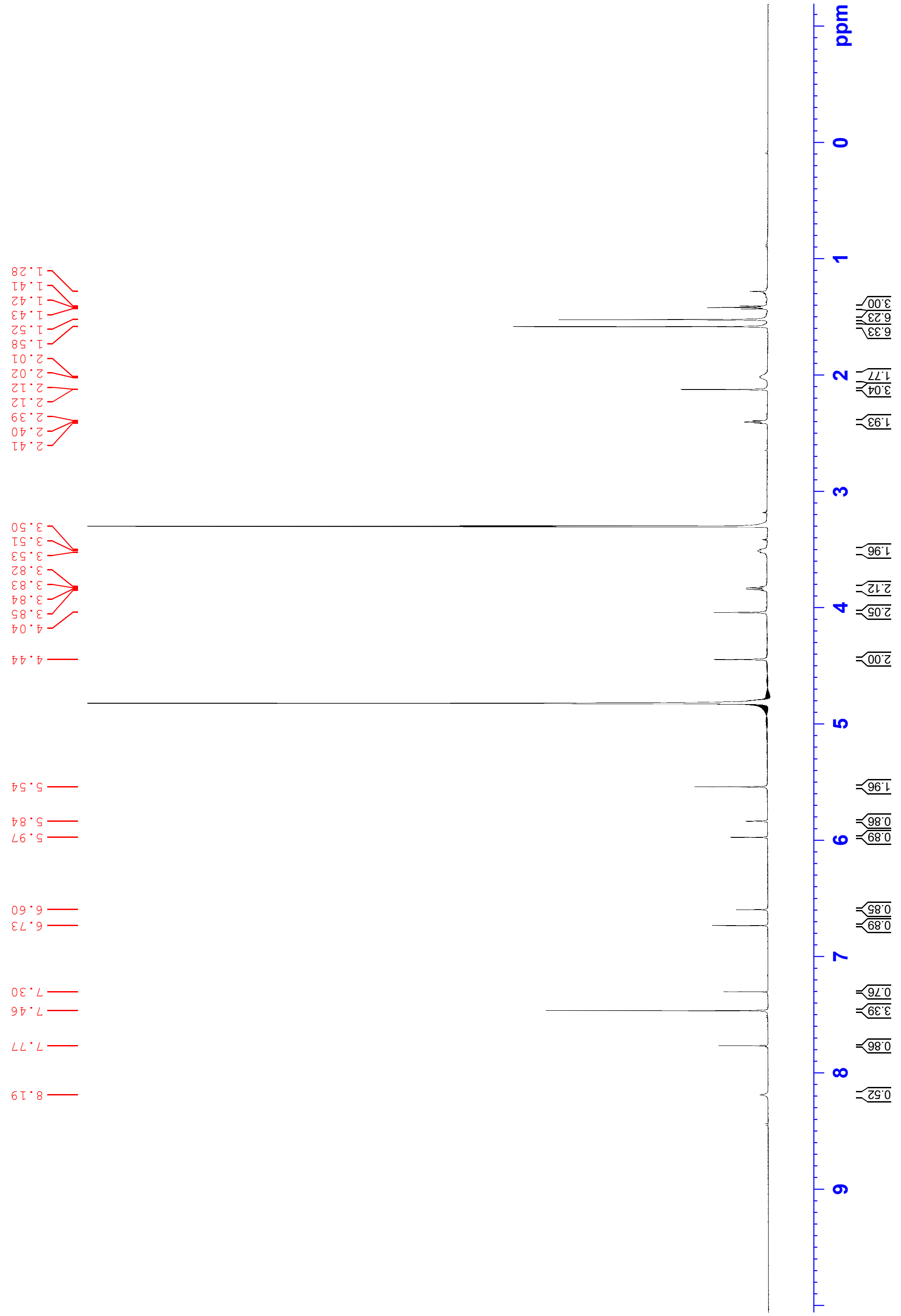

3.2. (11-(4-((2-(2-((6-Chlorohexyl)oxy)ethoxy)ethyl)amino)-4-oxobutyl)-1-ethyl-2,2,4,10,10-pentamethyl-10,11-dihydro-2H-dipyrido[3,2-b:2',3'-i]phenoxazin-1-ium-8-yl)methanesulfonate (CA-ATTO700)

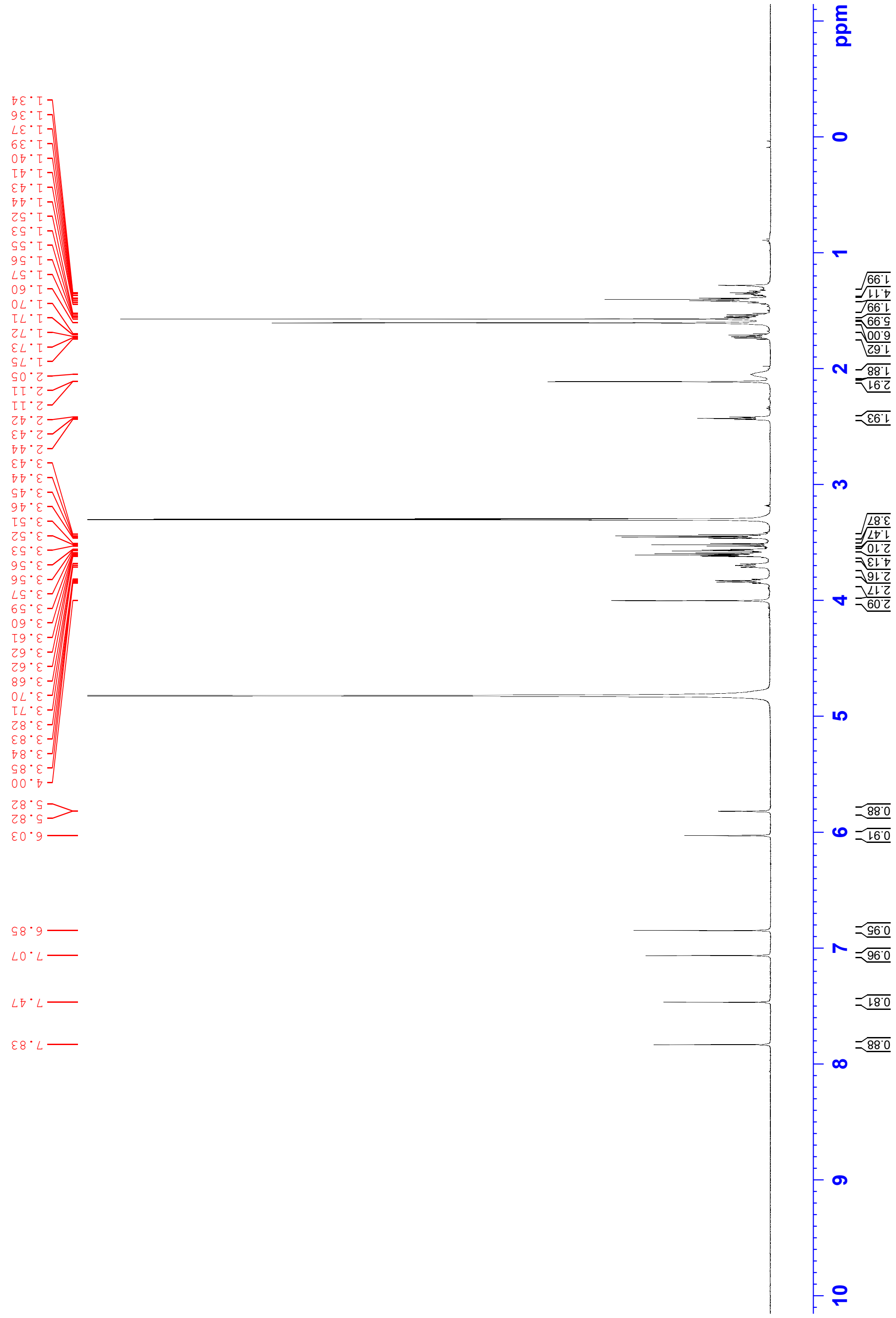

4. Protein labelling

4.1. SNAP–Halo construct[2]

SNAP–Halo sequence:

```
MASWSHPQFE KGADDDDKVP HMDKDCEMKR TTLDSPLGKL ELSGCEQGLH EIIFLGKGTS
AADAVEVPAP AAVLGGPEPL MQATAWLNAY FHQPEAIEEF PVPALHHPVF QQESFTRQVL
WKLLKVVKFG EVISYSHLAA LAGNPAATAA VKTALSGNPV PILIPCHRVV QGDLDVGGYE
GGLAVKEWLL AHEGHRLGKP GLGGRLEVLF QGPKAFLEGS EIGTGFPFDP HYVEVLGERM
HYVDVGPRDG TPVLFLHGNP TSSYVWRNII PHVAPTHRCI APDLIGMGKS DKPDLGYFFD
DHVRFMDAFI EALGLEEVVL VIHDWGSALG FHWAKRNPER VKGIAFMEFI RPIPTWDEWP
EFARETFQAF RTTDVGRKLI IDQNVFIEGT LPMGVVRPLT EVEMDHYREP FLNPVDREPL
WRFPNELPIA GEPANIVALV EEYMDWLHQS PVPKLLFWGT PGVLIPPAEA ARLAKSLPNC
KAVDIGPGLN LLQEDNPDLI GSEIARWLST LEISGAPGFS SISAHHHHHH HHHH*
```

Strep-Tag II, Enterokinase-site, SNAP, Precission Sequence, Halo, His-Tag

4.2. SNAP–Halo expression and purification

SNAP-Halo with an N-terminal Strep-tag and C-terminal 10xHis-tag was cloned into a pET51b(+) expression vector for bacterial expression was expressed and purified as described previously.[2]

4.3. Protein labelling

4.3.1. For turn-on measurements

<u>Absorbance:</u>
Labelling substrates CA-ATTO700 and BG-ATTO700 were dissolved in DMSO to a concentration of 1 mM and diluted in activity buffer (containing: 50 mM NaCl, 50 mM HEPES, pH = 7.3 + 4 µg/mL BSA) to 20 µM. 5 µL of each dye solution were incubated for 1 h at r.t. with 5 µL of 82 µM SNAP-Halo Protein diluted in activity buffer. Reaction mixes were recorded on a Nanodrop 2000C in triplicates. Data plotting was performed in GraphPad Prism 8.

<u>Quantum yields:</u>
Labelling substrates CA-ATTO700 and BG-ATTO700 were dissolved in DMSO to a concentration of 1 mM and diluted in activity buffer (containing: 50 mM NaCl, 50 mM HEPES, pH = 7.3 + 4 µg/mL BSA) to 500 nM. 3 mL of each dye solution were incubated for 1 h at r.t. with 37 µL of 82 µM SNAP-Halo Protein. Reaction mixes were transferred into cuvettes and quantum yields were recorded on a HAMAMATSU QuantaurusQY in triplicates (ATTO700: λ_{Ex} = 660 nm; λ_{Em} = 716 nm). Data plotting was performed in GraphPad Prism 8.

<u>Fluorescence:</u>
Labelling substrates CA-ATTO700 and BG-ATTO700 were dissolved in DMSO to a concentration of 1 mM and diluted in activity buffer (containing: 50 mM NaCl, 50 mM HEPES, pH = 7.3 + 4 µg/mL BSA) to 400 nM. 100 µL of each dye solution were incubated for 1 h at r.t. with 100 µL of 2 µM SNAP/Halo/SNAP-Halo Protein diluted in activity buffer. Reaction mixes were transferred into Greiner black flat bottom 96 well plates and excitation/emission profiles were recorded on a TECAN INFINITE M PLEX plate reader as quadruplicates (ATTO700: λ_{Ex} = 650±20 nm; λ_{Em} = 780±20 nm; 10 flashes; 40 µs integration time). Data normalization, integration and plotting was performed in GraphPad Prism 8.

4.3.2. For FRET measurements

Labelling substrates BG-JF_{646}, BG-SiR-d12, BG-Sulfo646, BG-SulfoCy5 and CA-ATTO700 were dissolved in DMSO to a concentration of 1 mM and diluted in activity buffer to 10 µM. SNAP-Halo protein was diluted in activity buffer to a concentration of 2 µM. 280 µL of protein solution were incubated for 1 h at r.t. with 140 µL BG-dye mix and 140 µL CA-dye mix or with 280 µL of each single dye mix or 280 µL activity buffer. To remove surplus of unbound dye, His-tag affinity chromatography was performed. 50 µL of Ni-NTA beads were transferred into an Eppendorf tube and washed two times with 500 µL of washing buffer (containing: 25 mM Na_2HPO_4, 25 mM NaH_2PO_4, 300 mM NaCl, 15 mM Imidazole, pH = 7). Protein-dye mix was incubated with washed beads for 30 min at r.t. on a rotator. Supernatant was removed and beads were washed 5 times with 500 µL washing buffer for 5 min. Finally, protein was eluted in 100 µL elution buffer (containing: 25 mM Na_2HPO_4, 25 mM NaH_2PO_4, 300 mM NaCl, 500 mM imidazole, pH = 7). 20 µL of each labelled protein was transferred into Greiner black flat bottom 384 well plate and emission/excitation profiles were recorded on a TECAN INFINITE M PLEX plate reader (λ_{Ex} = 560±20 nm; λ_{Em} = 600–850±20 nm; 10 flashes; 40 µs integration time). Data normalization, integration and plotting was performed in GraphPad Prism 8. For QTOF-MS measurements, 20 µL of each purified labelled protein was transferred into a mass-spec vial before full protein mass was acquired. The remaining purified proteins were diluted in 50% Glycerol, snap-freezed and stored at -80°C till further usage.

4.4. SNAP–Halo mass spectrometry[3]

Condition	calc.	found
SNAP–Halo	59072	59070
SNAP:ATTO700–Halo	59739	59738
SNAP–Halo:ATTO700	59807	59806
SNAP:Sulfo646–Halo	59971	59971
SNAP:SulfoCy5–Halo	59815	59814
SNAP:JF_{646}–Halo	59669	59669
SNAP:SiR-d12–Halo	59657	59655
SNAP:Sulfo646–Halo:ATTO700	60706	60706
SNAP:SulfoCy5–Halo:ATTO700	60551	60546
SNAP:JF_{646}–Halo:ATTO700	60404	60404
SNAP:SiR-d12–Halo:ATTO700	60392	60389

5. SNAP26m-TM-Halo Cloning

5.1. Construct

SNAP26m–TM–Halo sequence:

```
METDTLLLWV LLLWVPGSTG DYPYDVPDYA GAQPARSDKD CEMKRTTLDS PLGKLELSGC
EQGLHEIKLL GKGTSAADAV EVPAPAAVLG GPEPLMQATA WLNAYFHQPE AIEEFPVPAL
HHPVFQQESF TRQVLWKLLK VVKFGEVISY QQLAALAGNP AATAAVKTAL SGNPVPILIP
CHRVVSSSGA VGGYEGGLAV KEWLLAHEGH RLGKPGLGVD EQKLISEEDL NAVGQDTQEV
IVVPHSLPFK VVVISAILAL VVLTIISLII LIMLWQKKPR GAQPARSGSE IGTGFPFDPH
YVEVLGERMH YVDVGPRDGT PVLFLHGNPT SSYVWRNIIP HVAPTHRCIA PDLIGMGKSD
KPDLGYFFDD HVRFMDAFIE ALGLEEVVLV IHDWGSALGF HWAKRNPERV KGIAFMEFIR
PIPTWDEWPE FARETFQAFR TTDVGRKLII DQNVFIEGTL PMGVVRPLTE VEMDHYREPF
LNPVDREPLW RFPNELPIAG EPANIVALVE EYMDWLHQSP VPKLLFWGTP GVLIPPAEAA
RLAKSLPNCK AVDIGPGLNL LQEDNPDLIG SEIARWLSTL EISGVD*
```

Igk leader, HA tag, SNAP, Myc Tag, PDGFR TM, Halo

5.2. Primer

pDISPLAY_f	GTCGACGAACAAAAACTCATCTCAGAAGAGG
pDISPLAY_r	TGATCTGGCCGGCTGGGC
SNAP26m_f	gggcccagccggccagatcaGACAAAGACTGCGAAATGAAGCGCACC
SNAP26m_r	atgagtttttgttcgtcgacGCCCAGCCCAGGCTTGCC

5.3. Protocol

Replacing SNAP$_f$ with SNAP26m in our reported plasmid based on pDisplay was performed bythe Gibson assembly strategy. We amplified SNAP26m and Halo pDISPLAY with the indicated primers to obtain overlapping DNA fragments than then were ligated in a single iso-thermal reaction according to the manufacturer's instructions (NEB, #E5510).

6. Cell culture

6.1. Culture protocols

HEK293T cells were cultured in growth medium (DMEM, Glutamax, 4.5 g Glucose, 10% FCS, 1% PS; Invitrogen) at 37 °C and 5% CO_2. 30,000 cells/well were seeded on 8-well µL slides (Ibidi) previously coated with 0.25 mg/ml poly-L-lysine (Aldrich, mol wt 70000-150000). The next day, 400 ng DNA was transfected using 0.8 µL Jet Prime reagent in 40 µL Jet Prime buffer (VWR) per well. Medium was exchanged against antibiotic free media before the transfection mix was pipetted on the cells. After 4 hours incubation at 37 °C and 5% CO_2, medium was exchanged against growth media. After 24 hours cells were stained and imaged. All dyes were used in a concentration of 100 nM.

6.2. WST-1 viability assay

HEK293T cells were seeded (20,000 cells/well) in a clear 96 well-plate and allowed to grow for 2 days in 100 µL Fluorobrite medium supplemented with 10% FBS at 37 °C and 5% CO_2. Stock solutions of BG-ATTO700 and CA-ATTO700 were prepared and 50 µL was added on top of the cells, allowing to incubate for 2 hours. The medium was carefully removed and replaced by WST-1 (#MK400, Takara Bio) containing Fluorobrite medium supplemented with 10% FBS according to the manufacturer's instructions. Incubation was performed for another 2 hours before absorbance was read on a TECAN INFINITE M PLEX plate reader (λ_{Abs} = 440 nm) and corrected by substraction ($\lambda_{Abs\ correct}$). Data points were normalized to non-treated cells, and wells containing no cells were used as controls. Plotting was performed in GraphPad Prism 8. were read

7. Microscopy

7.1. Widefield, confocal and STED

Widefield imaging was performed on a TIE Nikon epifluorescence microscope equipped with a pE4000 (cool LED), Penta Cube (AHF 66-615), 60x oil NA 1.49 (Apo TIRF Nikon) and a sCMOS camera (Prime 95B, Photometrics) operated by NIS Elements (Nikon). For excitation the following LED wavelengths were used: Hoechst: 405 nm; JF_{549} and Sulfo549: 550 nm; ATTO700: 635 nm.
Confocal and live STED microscopy on transfected SNAP-GLP1R:HEK293 and Halo-GLP1R:HEK293 was performed using a Leica SP8 TCS STED FALCON (Leica Microsystems) equipped with a pulsed white-light excitation laser (80 MHz repetition rate, NKT Photonics), a 100x objective (HC PL APO CS2 100×/1.40 NA oil), a temperature-controlled chamber and operated by LAS X. JF_{646} was excited using λ = 640 nm and emission signals were captured at λ = 650–750 nm. ATTO700 stained cells were excited using λ = 670 nm and emission signals were captured at λ = 680–750 nm. For Live STED images 775 nm depletion of 40-60% for JF_{646} and 10-15% for ATTO700 and 16x line averaging were used. Both confocal and STED images were collected using a time gated Hybrid detector (0.5–6 ns). Images of 1024x1024 pixel had a pixel size of 18.9 nm. Line profiles of 20pxl width (189 nm) of closely opposed plasmamembrane were selected and sigma and full wide at half maximum (FWHM, FWHM = 2.35 * sigma) were determined after Gaussian fitting (Image J). For saturation intensity and depletion efficiency, similar line scans as before were acquired, 775 nm power was measured with a powermeter (Thorlabs PM100A with S121C Sensor) and area of donut shaped depletion laser was determined from reflection signal of 150nm gold beads (Abberior).

7.2. Single molecule imaging, fluorescence life-time

The PicoQuant MicroTime 200 time-resolved fluorescence system based on an inverted Olympus IX73 microscope (Olympus, Tokyo, Japan) with a 60× Plan-Apo/1.4-NA water-immersion objective using a 560 nm and 690 nm excitation laser was used. For fluorescence life-time experiments, measurements were made of donor-only and donor-acceptor labeled constructs. SPAD signals were processed with the TimeHarp 300 photon counting board and analyzed with the SymPhoTime 64 software (PicoQuant, Berlin, Germany) taking into account the instrument response function to allow consideration of short lifetime components with a high accuracy. Fluorescent life-time of samples was acquired for 300 seconds, with a pixel integration time of 28 µs and an average photon count rate of 30,000 counts per second. Excitation light was split with a dichroic mirror at 488/561/685 nm and detected using a 620/60 band pass and 740/60 band pass for donor-only/donor-acceptor and acceptor-only constructs, respectively. The pinhole was set to 100 µm. Fluorescent lifetimes were obtained from TCSPC decay curves fitted by an exponential equation using the SymPhoTime 64 software. Fitting of fluorescence decays was performed with a single exponential model. By characterizing donor lifetime in the absence and presence of acceptor, FRET efficiency can be calculated from:

$$Eff_{FRET} = 1 - \frac{\tau_{DA}}{\tau_D}$$

where τ_{DA} and τ_D and are the donor excited state lifetime in the presence and absence of acceptor.

8. Supplementary Figures

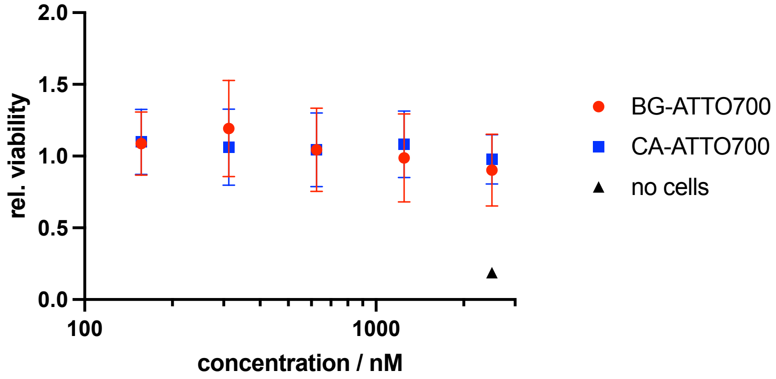

Supplementary Figure S1: Cell viability by WST-1 assay. HEK293 cells remain viable after 2 hour incubation with up to 2.5 uM of BG-ATTO700 or CA-ATTO700. No cells served as control, and values of relative viability are obtained by dividing by vehicle treatment values.

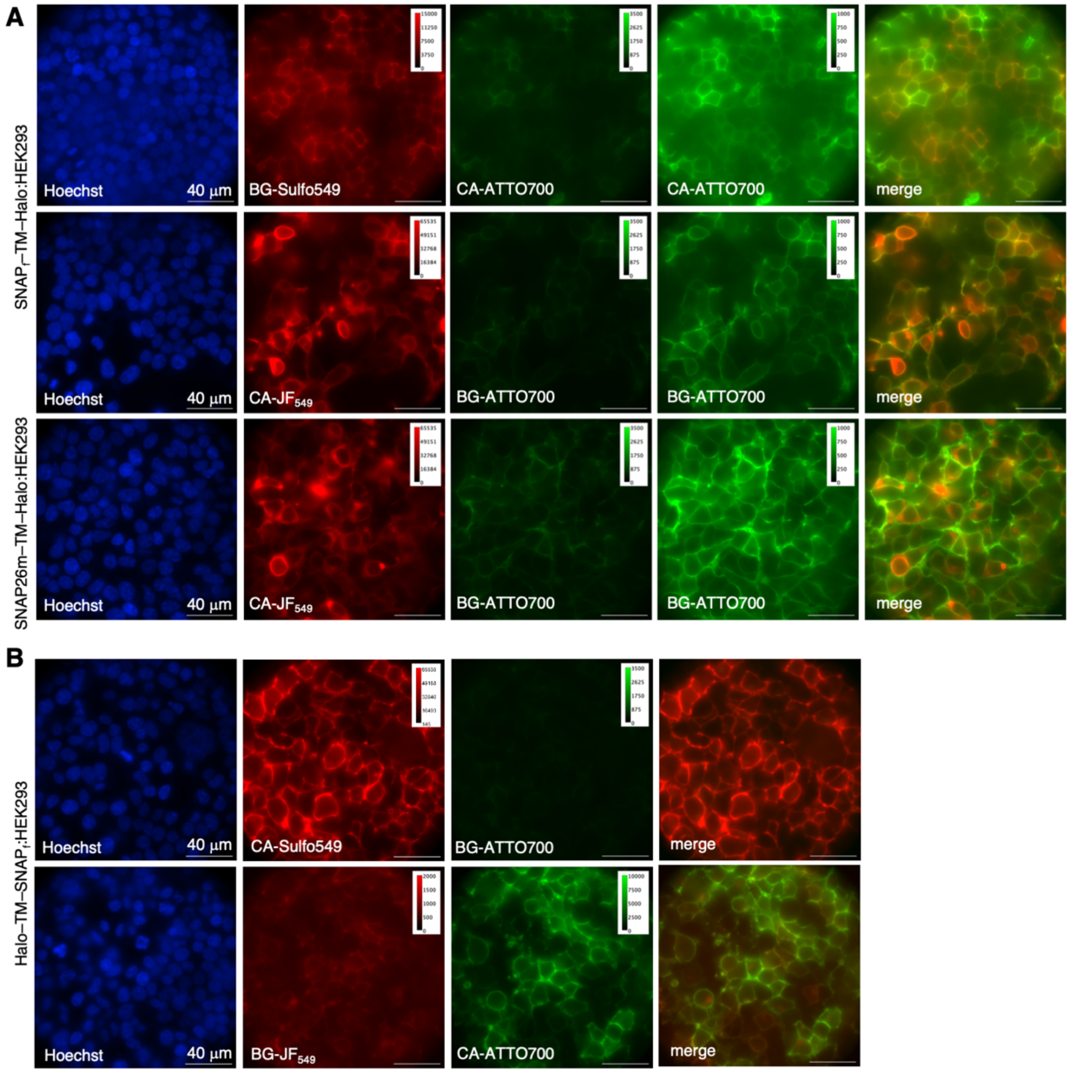

Supplementary Figure S2: Live cell microscopy. A) Widefield imaging of live SNAP$_f$-TM-Halo:HEK293 SNAP26m-TM-Halo:HEK293 cells incubated with either CA-ATTO700 and BG-Sulfo549 (top) or BG-ATTO700 and CA-JF$_{549}$ (bottom). **B**) Widefield imaging of live Halo-TM-SNAP:HEK293 cells incubated with either BG-ATTO700 and CA-Sulfo549 (top) or CA-ATTO700 and BG-JF$_{549}$ (bottom). Scale bars = 40 µm.

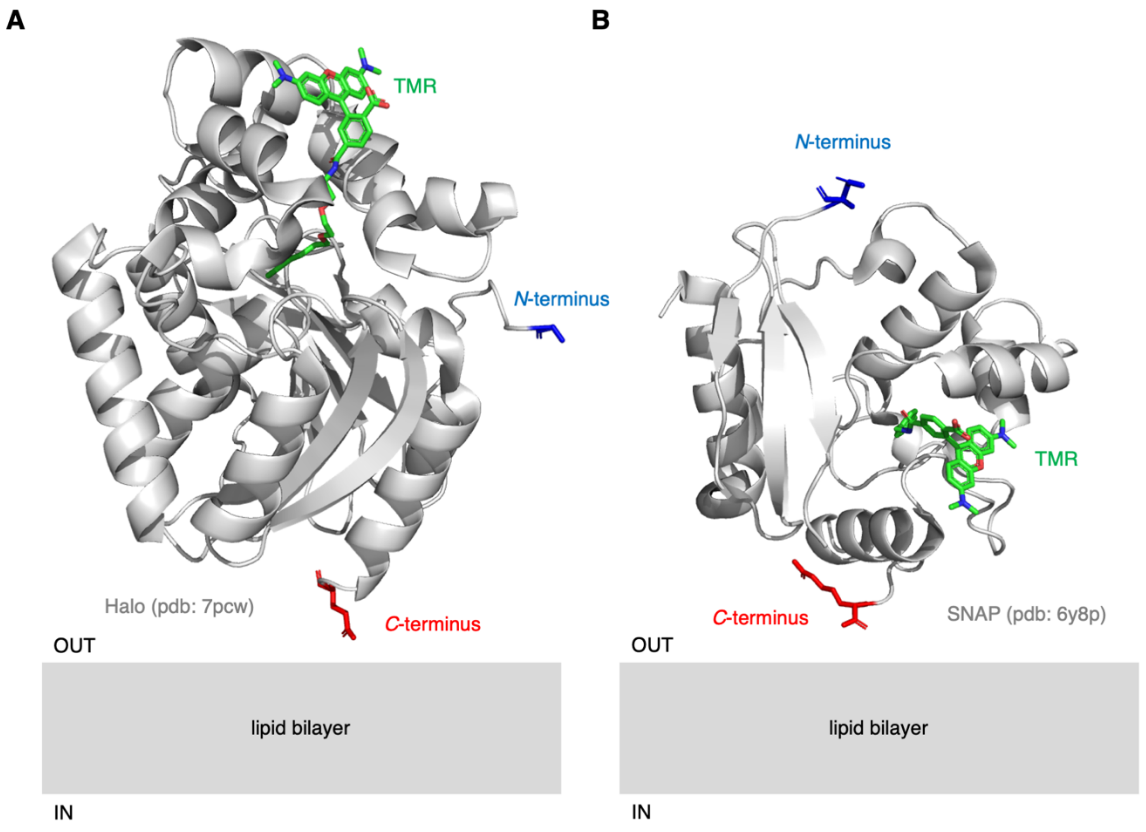

Supplementary Figure S3: Structural comparison of SNAP and Halo. Consulting the TMR-bound crystal structures of Halo and SNAP, the entry channel is considerably further away from the C-terminus for Halo (pdb: 7pcw)[4] (**A**) when compared to SNAP (pdb: 6y8p)[5] (**B**). Given that the membrane is negatively charged, this might lead to more repulsion of the negatively charged ATTO700. In turn, this may account for weaker signals when labelling a SNAP-TM-Halo construct in live cells.

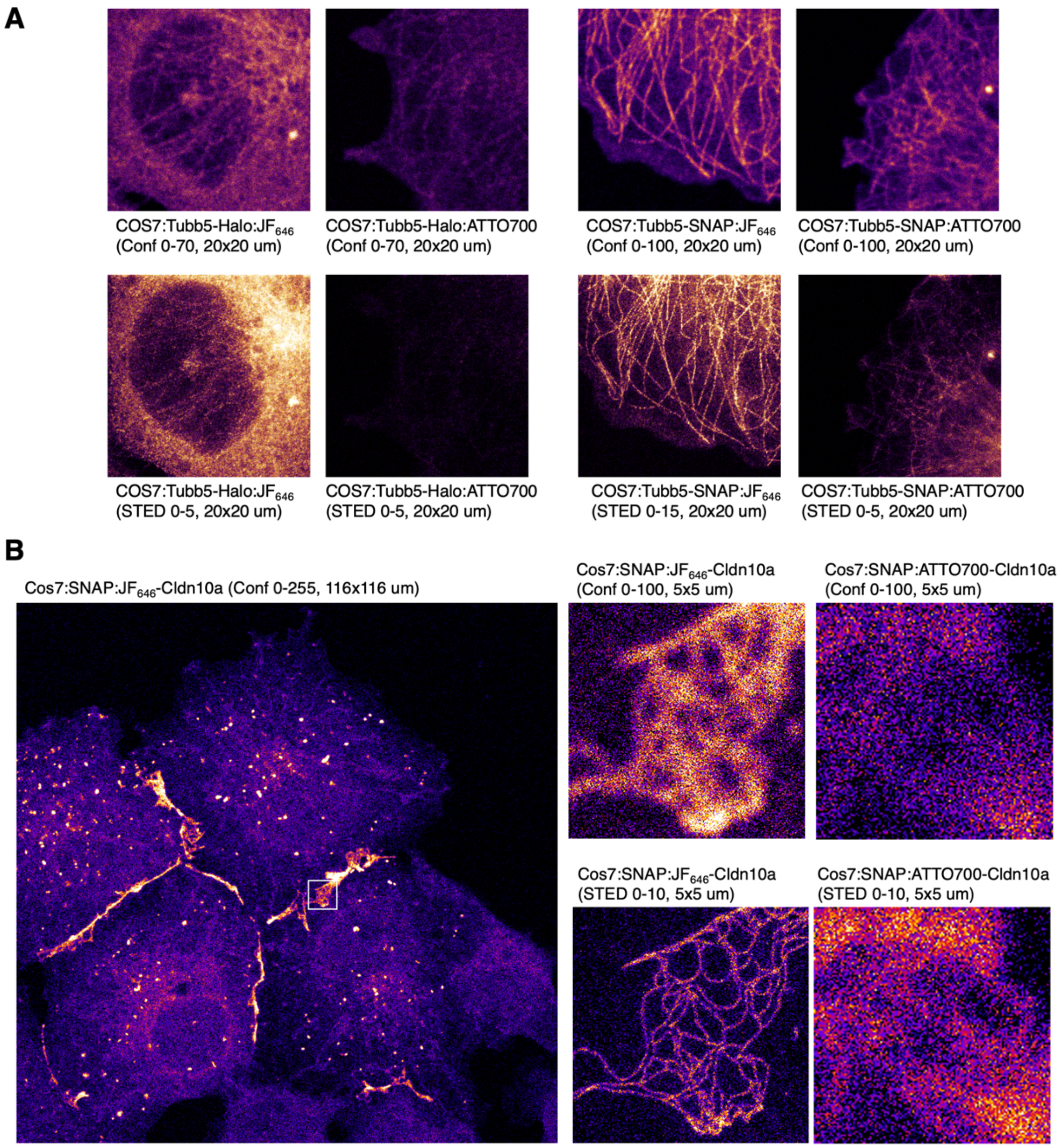

Supplementary Figure S4: STED imaging of tubulin and claudin networks. Cos7 cells were transfected with Tubb5-SNAP / Tubb5-Halo (**A**) or SNAP-Cldn10a (**B**) before fixation and labelling with the JF_{646} or ATTO700.

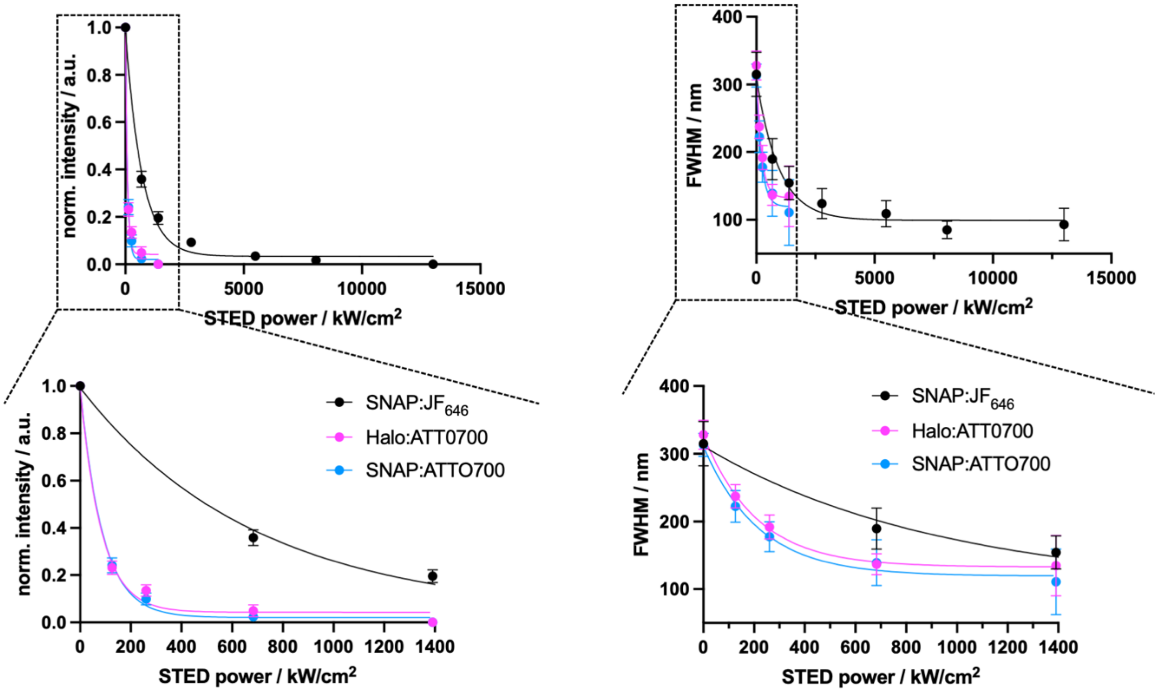

Supplementary Figure S5: Saturation intensity and depletion efficiency are plotted in full, with inserts taken from Figure 3H and 3I.

9. Supplementary Schemes

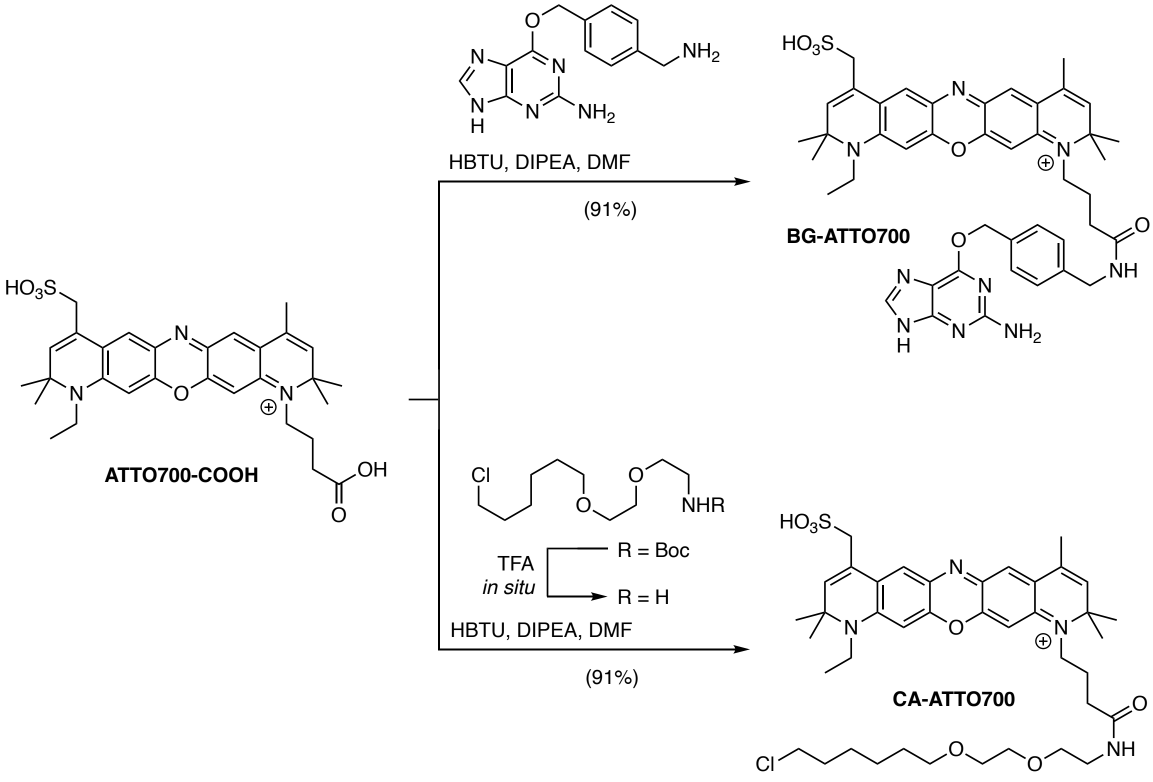

Supporting Scheme 1: Chemical synthesis BG-ATTO700 and CA-ATTO700.

10. References

1. Farrants, H. *et al.* SNAP-Tagged Nanobodies Enable Reversible Optical Control of a G Protein-Coupled Receptor *via* a Remotely Tethered Photoswitchable Ligand. *ACS Chem. Biol.* **13**, 2682–2688 (2018).
2. Roßmann, K. *et al.* N-Methyl deuterated rhodamines for protein labelling in sensitive fluorescence microscopy. *Chem Sci* **13**, 8605–8617 (2022).
3. Birke, R. *et al.* Sulfonated red and far-red rhodamines to visualize SNAP- and Halo-tagged cell surface proteins. *Org Biomol Chem* **20**, 5967–5980 (2022).
4. Frei, M. S. *et al.* Engineered HaloTag variants for fluorescence lifetime multiplexing. *Nat Methods* **19**, 65–70 (2022).
5. Wilhelm, J. *et al.* Kinetic and Structural Characterization of the Self-Labeling Protein Tags HaloTag7, SNAP-tag, and CLIP-tag. *Biochemistry* **60**, 2560–2575 (2021).
